# The predictive value of diaphragm ultrasound for weaning outcomes in critically ill children

**DOI:** 10.1186/s12890-019-1034-0

**Published:** 2019-12-30

**Authors:** Yang Xue, Zhen Zhang, Chu-Qiao Sheng, Yu-Mei Li, Fei-Yong Jia

**Affiliations:** 1 Department of Developmental and Behavioral Pediatrics, The First Hospital of Jilin University, 71 Xinmin Street, Changchun, 130021 China; 2 Department of Pediatrics Intensive Care Unit, The First Hospital of Jilin University, Changchun, China

**Keywords:** Paediatric, Mechanical ventilation, Diaphragm, Ultrasound, Weaning, Pimax

## Abstract

**Introduction:**

Multiple studies have shown that diaphragmatic ultrasound can better predict the outcome of weaning in adults. However, there are few studies focusing on children, leading to a lack of sufficient clinical evidence for the application of diaphragmatic ultrasound in children. The purpose of this study was to investigate the predictive value of diaphragm ultrasound for weaning outcomes in critically ill children.

**Methods:**

The study included 50 cases whose mechanical ventilation (MV) time was > 48 h, and all eligibles were divided into either the weaning success group (*n* = 39) or the weaning failure group (*n* = 11). Diaphragm thickness, diaphragmatic excursion (DE), and diaphragmatic thickening fraction (DTF) were measured in the zone of apposition. The maximum inspiratory pressure (PImax) was also recorded.

**Results:**

The ventilatory treatment time (*P* = 0.002) and length of PICU stay (*P* = 0.013) in the weaning failure group was longer than the success group. Cut-off values of diaphragmatic measures associated with successful weaning were ≥ 21% for DTF with a sensitivity of 0.82 and a specificity of 0.81, whereas it was ≥0.86 cm H_2_O/kg for PImax with a sensitivity of 0.51 and a specificity of 0.82. The linear correlation analysis showed that DTF had a significant positive correlation with PImax in children (*P* = 0.003).

**Conclusions:**

Diaphragm ultrasound has potential value in predicting the weaning outcome of critically ill children. DTF and PImax presented better performance than other diaphragmatic parameters. However, DE has limited value in predicting weaning outcomes of children with MV.

**Trial registration:**

Current Controlled Trials ChiCTR1800020196, (Dec 2018).

## Background

Mechanical ventilation (MV) technology is widely used in paediatric critical care. About 30% of children in the paediatric intensive care unit (PICU) receive MV support [1]. However, MV support is not the end of the treatment, and the ultimate goal is to help patients wean off of MV support. An international consensus conference on weaning from MV in 2007 proposed that weaning should be categorized into three groups: simple weaning, difficult weaning, and prolonged weaning [2]. A multicentre study has shown that 10% of patients with MV had a difficult weaning duration of more than 1 day and less than 1 week, and 9% had a prolonged weaning duration of 1 week or more [3]. Failure to wean (FTW) is generally defined as difficult and prolonged weaning. FTW has significantly worse clinical outcomes. Studies have shown that FTW is an independent risk factor for mortality in ICU patients and prolonged length of ICU stay, and it is also associated with the occurrence of intensive care unit-acquired weakness and ventilator-induced diaphragmatic dysfunction [4–6]. Therefore, weaning from mechanical ventilation represents a crucial step for every patient. The optimal timing of weaning can shorten the duration of MV and reduce complications. Weaning predictors – such as rapid shallow breathing index, airway occlusion pressure 0.1 s, maximum inspiratory pressure (PImax), and the weaning index – have been used to improve the rate of successful weaning in adult studies [7–9]. Unfortunately, in terms of weaning success in children, there is an insufficient amount of data to suggest the usefulness of predictors being superior to clinical judgment [10].

As a new technology in recent years, diaphragm ultrasound allows for the direct visualisation of the diaphragmatic function of patients [11–13], which has the advantages of being noninvasive, rapid, and easy to perform at the bedside. Therefore, it is suitable for application in patients with MV in ICU [12, 13]. Multiple adult studies have shown that diaphragmatic ultrasound can better predict the outcome of weaning, which has great value on guiding weaning in patients with MV [14–16]. However, there are few studies of diaphragmatic ultrasound in the field of paediatric critical medicine, leading to diaphragmatic ultrasound data being insufficient. In addition, the respiratory physiology and anatomical characteristics of children are different from that of adults. Therefore, the conclusions of adult studies may not be applicable to children, and more studies in children are needed to confirm the effectiveness of diaphragmatic ultrasound in predicting the outcome of weaning. This paper is the first study to investigate the predictive value of diaphragm ultrasound for weaning outcomes in critically ill children**.**

## Methods

### Patients

This prospective study was conducted in the paediatric intensive care unit of First Hospital of Jilin University, Changchun, China. Study subjects included 61 consecutive patients between January 2019 and May 2019, who were aged less than 18 years. The institutional ethics committee of the hospital approved the study protocol (ChiCTR1800020196). The parents or guardians of the eligible children provided written informed consent. An information sheet was provided for the parents or guardians of the participants.

All children who received MV support for ≥48 h and met the standard criteria for weaning readiness (improvement in the cause of primary disease, P_aO2_/F_iO2_ > 200, positive end-expiratory pressure (PEEP) ≤ 5–10 cm H_2_O, F_iO2_ ≤ 50%, and hemodynamically stable in the absence of vasopressors) were included in the study [17]. If the child experienced a known neuromuscular disease (such as amyotrophic lateral sclerosis, Guillain-Barre, or myasthenia gravis), cervical spinal cord injury, pneumothorax, death during mechanical ventilation, or if there was an unwillingness of the parents or guardians to participate in the study, then that child was excluded from the study.

### Study design

All eligibles underwent the spontaneous breathing test (SBT), which was performed using pressure support trials with a pressure support (8 cm H_2_O) and 5 cm H_2_O PEEP using a Drager Evita 4 ventilator for 30 min. Patients who were unable to tolerate spontaneous breathing tests during observation time were classified as failed weaning [2]. Ultrasound measurements and PImax were taken at the fifth minute after the start of SBT. The patient passed the SBT if the exhaled tidal volume was equal to or above 5 mL/kg of the ideal body weight, and if the respiratory rate remained within the targeted range for age (< 6 months, 20–50 breaths/min; 6 months-2 yr., 15–45 breaths/min; 2–5 yr., 15–40 breaths/min; > 5 yr. 10–35 breaths/min) [18]. All patients accepted the Venturi inside the mask for oxygen therapy after passing the SBT. Successful weaning was defined as the ability to maintain spontaneous breathing for > 48 h.

### PImax measurement

The measurement of PImax was occluding the airway at end expiration through a unidirectional valve, and maintained for approximately 10 breaths or 20 s [19]. Finally, the maximum negative pressure displayed by the ventilator was recorded. Body weight (BW) is known as a predictor of PImax in healthy children [20], therefore, the PImax was standardised by BW (PImax/BW).

### Diaphragm ultrasound measurement

**Fig. 1:**
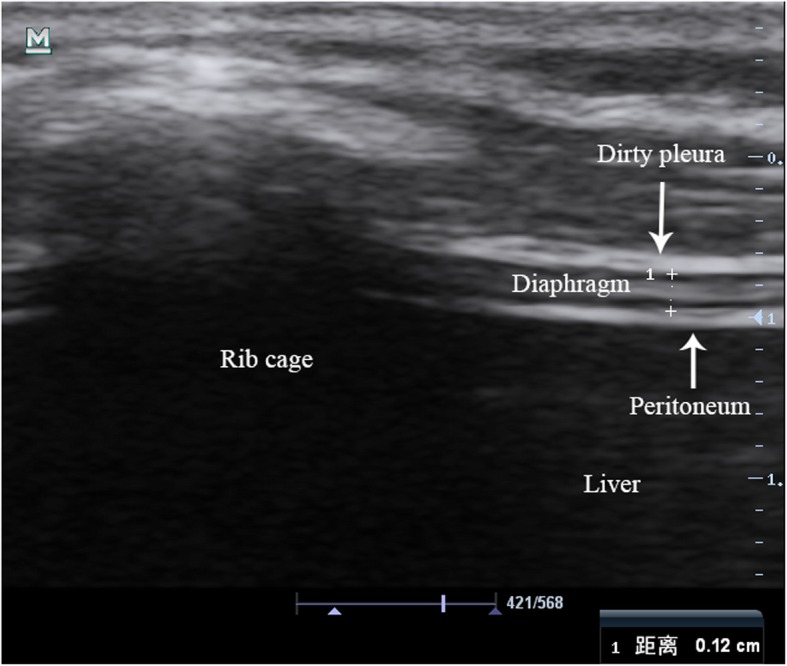
Ultrasound B-mode using a 10 MHz probe in the zone of apposition

**Fig. 2:**
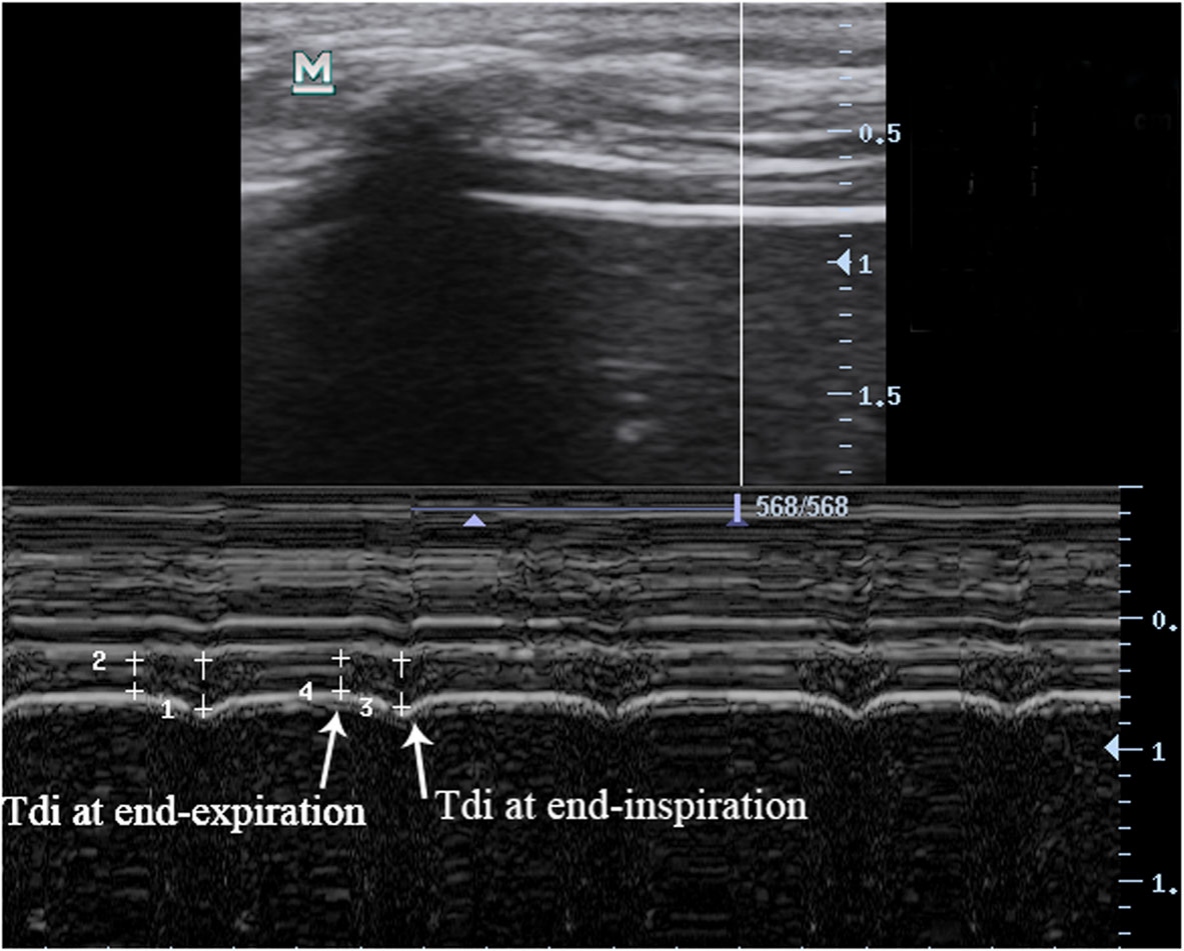
Ultrasound M-mode using a 10 MHz probe in the zone of apposition

All patients were placed in a semi-recumbent position with the head of the bed at a 30-degree angle. Two experienced sonographers performed ultrasound measurements by using the same portable ultrasound machine (Mindray, M7 series, China), and the evaluators were blinded to the results of the SBT prior to measurement. In the present study, only the right hemidiaphragm was measured because the right hemidiaphragm was more feasible and repeatable compared with the left hemidiaphragm [21]. Diaphragm thickness (Tdi) was measured by using a 10 MHz linear probe at the zone of apposition at the right eighth or ninth intercostal space, which is between the anterior axillary and the midaxillary lines. The direction of the ultrasound probe was perpendicular to the diaphragm. At this position, the diaphragmatic ultrasound image was a hypoechoic structure between two echoic lines (the diaphragmatic pleura and the peritoneal membrane) in the B-mode (Fig. 1). In the same position, M-mode ultrasonography was used to measure resting Tdi at end-expiration (Tdi-exp) and end-inspiration (Tdi-insp), respectively (Fig. 2). The Tdi measurement was the inner edge of the peritoneal membrane to the inner edge of the diaphragmatic pleura. The calculation formula of diaphragmatic thickening fraction (DTF) was (Tdi-insp – Tdi-exp) / Tdi-exp.

**Fig. 3:**
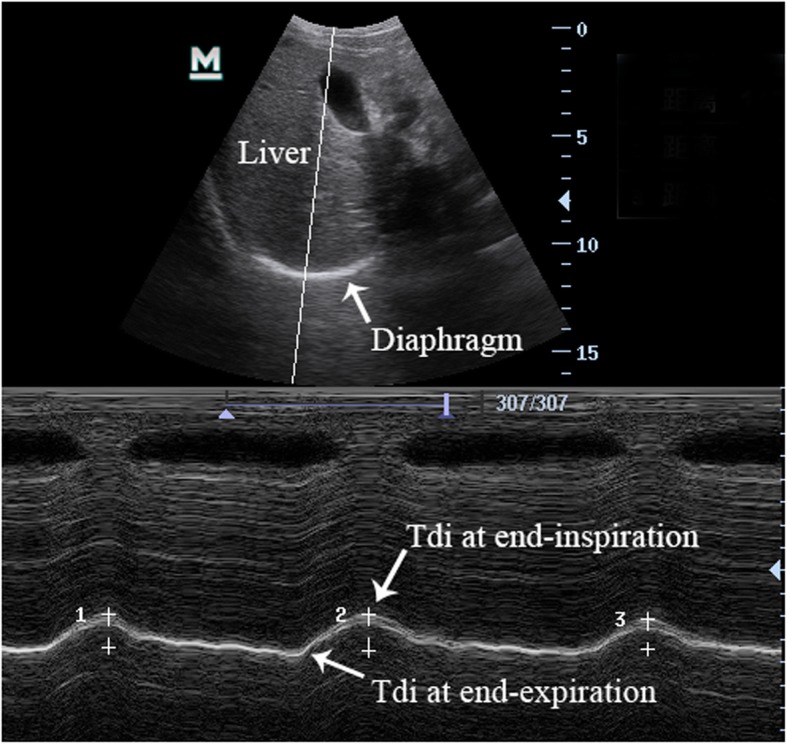
Ultrasound assessment of diaphragm diaphragmatic excursion in M-mode

For the measurement of diaphragmatic excursion (DE), a 5 MHz probe was placed at the junction of the right mid-clavicle line and the right subcostal margin, where the probe direction paralleled the diaphragmatic movement. The diaphragmatic movement toward the probe during inspiration was recorded as an upward motion of the M-mode tracing, and the movement was opposite during expiration. In a breathing cycle, the amplitude of DE was the maximum point that moved vertically downward to the lowest point in M-mode (Fig. 3) [22, 23]. The DE was continuously measured for 3 times in free breathing, and then the average was taken. DE and BW have significant positive correlations in children [24]. Therefore, DE was standardised by BW (DE/BW).

### Statistical analysis

Analyses were carried out using IBM SPSS Statistics for Windows, Version 22.0 (IBM Corp, Armonk, NY). Depending on whether distribution was normal or non-normal, continuous variables were described as mean ± SD or median (interquartile range). Categorical variables were described as n(%). Continuous variables were compared with Student’s t-test or Mann-Whitney U test. Depending on sample size, categorical variables were compared with Chi-squared test or Fisher’s exact test. The correlation analyses were conducted using the spearman method to test the relationship between DTF, PImax, and DE. To determine the best cut off for DE, DTF and PImax to predict weaning success, the area under the receiver operating characteristic (ROC) curve was calculated. For all final comparisons, a *p*-value less than or equal to 0.05 was considered statistically significant.

## Results

### Sample characteristics

**Fig. 4:**
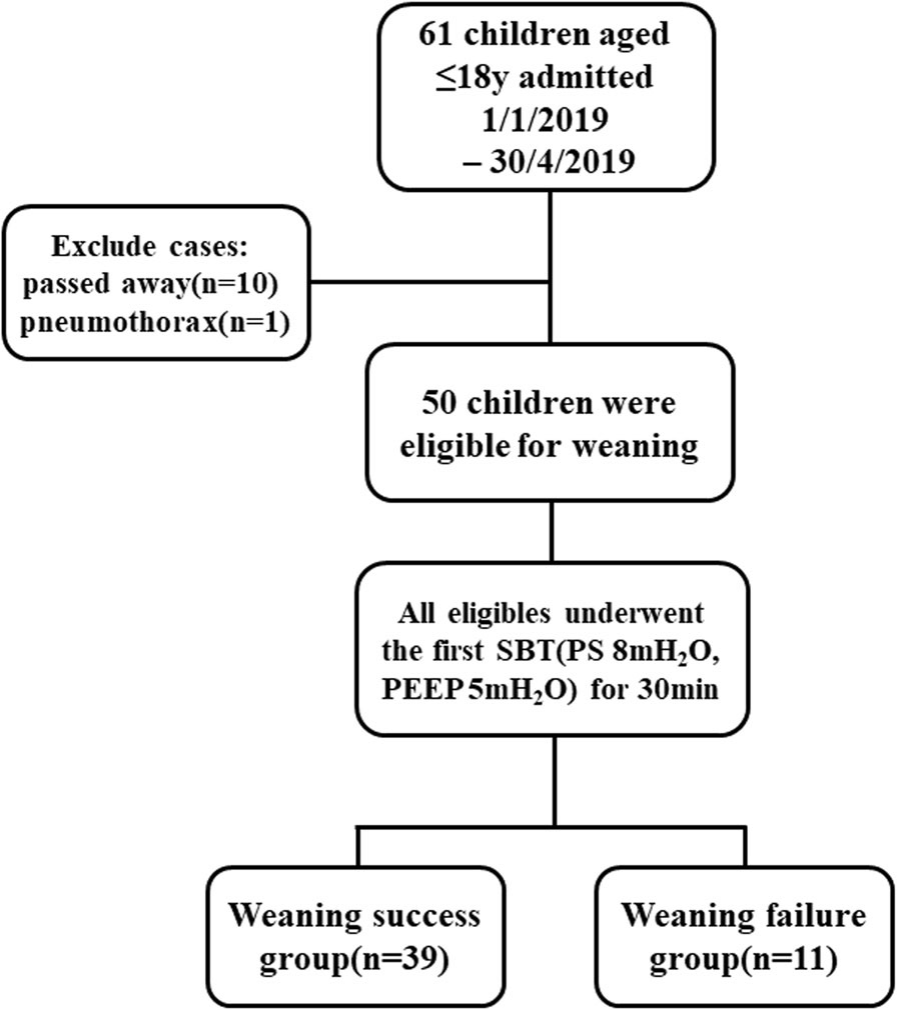
Flow chart of this study

Sixty-one patients underwent mechanical ventilation support during the study period. Eleven cases were excluded: ten cases passed away during mechanical ventilation, and one case had pneumothorax. Finally, 50 patients met the inclusion criteria. Eligibles were divided into either the weaning success group (*n* = 39) or weaning failure group (*n* = 11) (Fig. 4).

All patient characteristics are summarized in Table 1. Ventilatory treatment time (*P* = 0.002) and length of PICU stay (*P* = 0.013) in the weaning failure group was significantly longer than the success group. Three cases passed away after 48 h of successful extubation in the failure group, the in-hospital mortality was 27.3% (3/11), and one died in the success group. The DTF (*P* < 0.001) and was significantly higher in the weaning success group than the failure group. However, it should be noted that there were no differences in Tdi, DE, or PImax between the weaning success and failure groups (Table 2).

**Table 1:** Sample Characteristics

Characteristics	Weaning success Group(*n* = 39)	Weaning failure Group(*n* = 11)	*P*
Age, months, median (IQR)	36.00(15.00–84.00)	42.00 (10.00–158.00)	0.55
Male sex (%)	66.67 (26/39)	72.73 (8/11)	0.70
Weight, kg, median (IQR)	15.00 (10.00–28.00)	14.00 (8.00–35.00)	0.94
Height, cm (mean ± SD)	103.87 ± 32.77	107.86 ± 33.99	0.73
Cause of respiratory failure, n (%)			
Respiratory dysfunction	59 (23/39)	36 (4/11)	
Cardiovascular dysfunction	5 (2/39)		
Other organ dysfunction	10 (4/39)	64 (7/11)	
Sepsis	8 (3/39)		
Postoperative	18 (7/39)		
PCIS (mean ± SD)	82.18 ± 7.86	83.27 ± 5.82	0.67
Laboratory findings (mean ± SD)			
pH	7.46 ± 0.04	7.44 ± 0.06	0.17
PaO2, mmHg	99.31 ± 29.81	86.78 ± 15.38	0.19
PaCO2, mmHg	36.84 ± 12.10	37.45 ± 6.78	0.87
P/F	315.11 ± 102.25	301.09 ± 68.93	0.61
Ventilatory treatment time, d, median (IQR)	5.00 (4.00–7.00)	15.00 (7.00–22.00)	0.002
Length of PICU stay, d, median (IQR)	12.00 (10.00–20.00)	28.00(15.00–30.00)	0.013
In hospital mortality(%)		27.27 (3/11)	
Ventilation setting			
Mode	SIMV+PSV	SIMV+PSV	
VT, ml/kg of ideal body weight (IQR)	6.40 (5.76–8.00)	6.40 (5.00–6.78)	0.33
PEEP, cmH_2_O	5	5	

*PCIS* Pediatric Critical Illness Score, *IQR* Interquartile range; *SD* Standard Deviation, *VT* Tidal volume

**Table 2:** Weaning indexs of all patients

Weaning indexs	Weaning success Group(n = 39)	Weaning failure Group(n = 11)	*P*
DTF%(mean ± SD)	30.93 ± 11.23	15.98 ± 6.65	**< 0.001**
DE, mm/kg (mean ± SD)	0.74 ± 0.75	0.45 ± 0.32	0.23
Tdi at end inspiration, mm/kg (mean ± SD)	1.07 ± 0.88	1.08 ± 0.62	0.97
Tdi at end expiration, mm/kg (mean ± SD)	0.79 ± 0.61	0.91 ± 0.49	0.59
PImax, cmH_2_O/kg (mean ± SD)	0.91 ± 0.56	0.56 ± 0.49	0.07

*DTF* Diaphragmatic thickening fraction, *DE* Diaphragmatic excursion, *Tdi* Diaphragm thickness

*PImax* Maximum inspiratory pressure

### Diaphragmatic parameters and PImax predict the value of weaning success

Of the 39 patients who were categorized as having successful weaning, 32 had a DTF of ≥21%. Of the 11 who failed weaning, 9 had a DTF < 21%. The resulting positive predictive value (PPV) and negative predictive value (NPV) was 94 and 56%, respectively. An ROC curve was used to assess the diagnostic accuracy of DTF, DE. A cut-off value of DTF ≥ 21% was associated with weaning success with a sensitivity of 82% and a specificity of 81% (Table 3). The area under the ROC curve for DTF was 0.89 (95% confidence interval [0.78 to 0.99]) (Additional file 1: Figure 1A). DE has limited value in predicting weaning success (*P* = 0.20). The area under the ROC curve for DE was 0.63 (95% confidence interval [0.43 to 0.83]).

**Table 3:** DTF and PImax

Parameters	Sensitivity(%)	Specificity(%)	PPV(%)	NPV(%)	AUC
DTF ≥ 21%	82	81	94	56	0.89
PImax≥0.86cmH_2_O/kg	51	82	91	32	0.70

*PPV* Positive predictive value, *NPV* Negative predictive value, *AUC* Area under curve

Twenty cases with PImax ≥0.86 cm H_2_O/kg in the 39 patients who exhibited successful weaning had a resulting PPV of 91%. 9 cases that had a PImax < 0.86 cm H_2_O/kg of the 11 who failed weaning had a resulting NPV of 32%. A cut-off value of PImax ≥0.86 cm H_2_O/kg was associated with weaning success with a sensitivity of 51% and a specificity of 82%(Table 3). and the area under the ROC curve for PImax it was 0.70 (95% confidence interval [0.52 to 0.88]) (Additional file 1: Figure 1B).

### Correlation analysis within DTF, PImax, and DE

A Spearman linear correlation analysis was performed within DTF, PImax, and DE (Additional file 2: Table S1). The results showed that DTF had significant correlation with PImax (*r* = 0.410, *P* = 0.003). The same analysis was also performed between DTF and DE. The results showed that there was significant correlation between DTF and DE (*r* = 0.380, *P* = 0.006).

## Discussion

This paper is the first study to investigate the predictive value of diaphragm ultrasound for weaning outcomes in critically ill children. The findings of this study demonstrate that the DTF of patients in the group of weaning failure were significantly lower than those in the successful group, which is consistent with the results of previous studies [21, 25, 26], indicating that the patients with weaning failure generally had diaphragmatic dysfunction. At the same time, the study also demonstrates that the duration of MV in the failed group was significantly longer than that in the successful group, suggesting that the prolonged MV had promoted the occurrence of diaphragmatic dysfunction. Respiratory muscle weakness in critically ill patients was associated with difficulty in weaning from mechanical ventilation [27]. Therefore, monitoring diaphragm function during SBT was important for predicting the outcomes of weaning.

Among the 50 patients within the present study, the rate of weaning success was 78% (39/50), and the rate of weaning failure was 22% (11/50), which was lower than the previous study (30%) [28]. The areas under the ROC curve of DTF and PImax for patients with weaning success were 0.89 and 0.70, respectively. An optimal cut-off value for predicting weaning success of DTF and PImax was 21% and 0.86 cm H_2_O/kg. The results of the present study showed that DTF ≥ 21% was associated with weaning success with a sensitivity of 0.82 and a specificity of 0.81, and PImax ≥0.86 cm H_2_O/kg was associated with weaning success with a sensitivity of 0.51 and a specificity of 0.82. Both have good value for predicting the weaning success of children, but the predictive value of DTF is better than that of PImax. A study by Ferrari established that a DTF of 36% predicted successful weaning in patients requiring long-term ventilator support [29]. Farghaly et al. have showed that DTF% ≥ 34.2% was associated with successful extubation [23]. The results of the above studies are all adults, and the results of the present study (DTF ≥ 21%) are lower than the above studies. The main reasons for consideration are as follows: the thickness and strength of human skeletal muscle fibres vary with age, and the diaphragm is a skeletal muscle that also conforms to this physiological change [24], thus the DTF of children will be less than that of adults. In addition, the majority of patients in adult studies were elderly chronic obstructive pulmonary disease (COPD) patients, whose diaphragmatic muscle fibres had a chronic oxidative remodelling process [30], leading to diaphragm compensatory ability weakness. Accordingly, more extensive contraction was needed to meet the ventilation.^[23. 29]^ Further, most patients for studies that focus on children suffered mostly from acute respiratory diseases such as severe pneumonia and laryngitis – whose diaphragm had no chronic oxidative remodelling process and had good compensatory ability. The above may be the main reasons for the differences found between the results of the present study and those of adults.

PImax is often used in the assessment of respiratory muscles, which can indirectly react to inspiratory muscle strength. The study conducted by Ferrari et al. [29] demonstrated that PImax was positively correlated with DTF in adults with mechanical ventilation (*r* = 0.71, *P* < 0.05). Ueki et al. [31] have provided a similar result (*r* = 0.82, *P* < 0.01). The present study has also found a significant linear correlation between DTF and PImax, and it should be noted that the PImax value should be standardised by BW. However, the predictive value of PImax in children was not better than in adults [32]. Since children undergoing MV are also more sedated than adults, children are less cooperative resulting in insufficient inspiratory effort during the assessment of PImax. In addition, PImax is the result of a combination of all inspiratory muscles, though the development of intercostal muscles and sternocleidomastoid muscles in children was immature [33]. The above factors will reduce the predictive value of PImax in children.

There are several limitations in this study. First, a relatively small population was studied. Especially since 11 patients failed to wean, the ability to predict weaning success within the present study was not sufficiently provided. Second, DE has better value of predicting weaning outcome in adults. However, in the present study, DE had limited value in predicting weaning outcomes, although the authors standardised DE by BW. This may due to the size of the sample. Third, because there is currently no reference value of DTF in children, it is unknown whether the initial diaphragmatic function of the enrolled children within the present study is abnormal or not. Finally, though diaphragmatic endurance is also important for weaning from mechanical ventilation, the present study only assesses the diaphragm muscle strength. Therefore, the diaphragmatic time-tension index and other indicators can be used to explore the relationship between diaphragmatic endurance and weaning outcome.

## Conclusion

Diaphragm ultrasound has potential value in predicting the weaning outcome of critically ill children. DTF and PImax showed better performance in predicting weaning outcomes than other diaphragmatic parameters. However, DE has limited value on predicting weaning outcomes in children with MV.

## Supplementary information

**Additional file 1: Figure** S**1 A** Area under receiving operating characteristic curve for DTF% to predict weaning success. The optimum cut-off value of DTF% was ≥21% with an AUC of 0.89 (95% CI [0.78 to 0.99]); S**1 B** Area under receiving operating characteristic curve for DE to predict weaning success. The optimum cut-off value of DE was ≥8.40 mm with an AUC of 0.77 (95% CI [0.64 to 0.91])

**Additional file 2: Table S1**. linear correlation between DTF and PImax, DE

## Data Availability

The datasets used and/or analyzed during the current study are available from the corresponding author on reasonable request.

## Abbreviations

AUC: Area under curve
BW: Body weight
COPD: Chronic obstructive pulmonary disease
DE: Diaphragmatic excursion
DTF: Diaphragmatic thickening fraction
IQR: Interquartile range
MV: Mechanical ventilation
NPV: Negative predictive value
PCIS: Pediatric critical illness score
PEEP: Positive end-expiratory pressure
PICU: Pediatric intensive care unit
PImax: Maximum inspiratory pressure
PPV: Positive predictive value
SBT: Spontaneous breathing test
SD: Standard Deviation
Tdi: Diaphragm thickness
VT: Tidal volume
