# Supplementary figures and images for: The predictive value of diaphragm ultrasound for weaning outcomes in critically ill children
Source: BMC Pulm Med. 2019 Dec 30;19:270. doi: 10.1186/s12890-019-1034-0 (PMC6937936; doi:10.1186/s12890-019-1034-0)

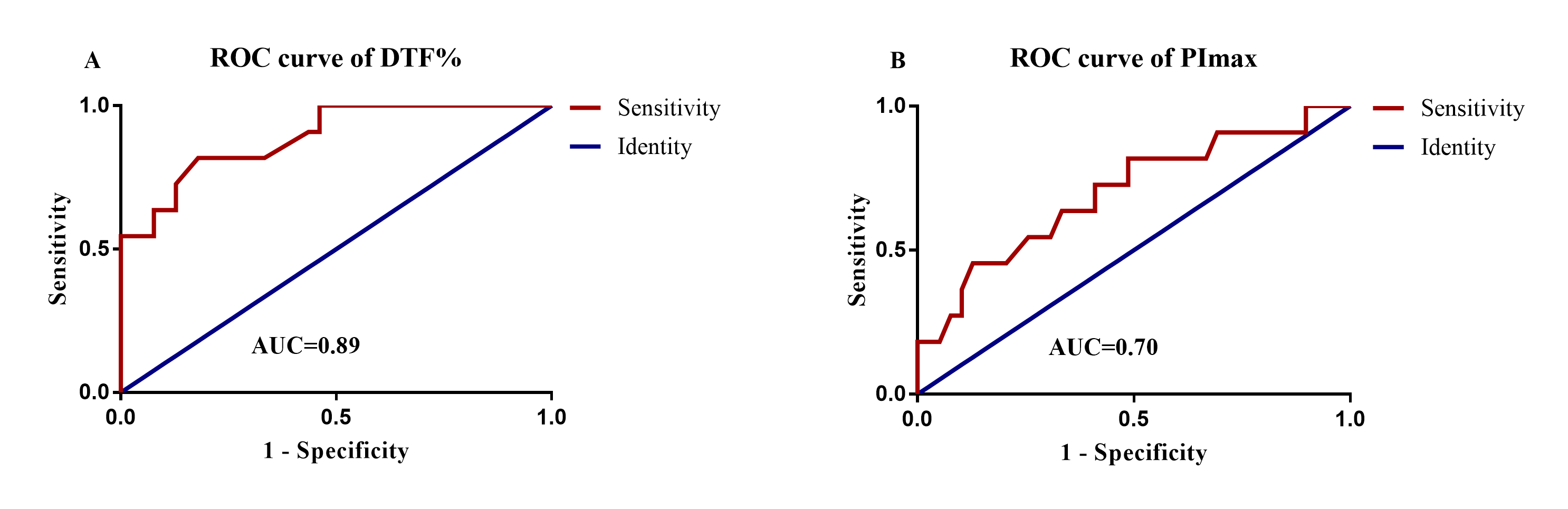

Supplement: Supplementary file 1 — Additional file 1: Figure S1 A Area under receiving operating characteristic curve for DTF% to predict weaning success. The optimum cut-off value of DTF% was ≥21% with an AUC of 0.89 (95% CI [0.78 to 0.99]); S1 B Area under receiving operating characteristic curve for DE to predict weaning success. The optimum cut-off value of DE was ≥8.40 mm with an AUC of 0.77 (95% CI [0.64 to 0.91]) [file 12890_2019_1034_MOESM1_ESM.tif]
