# Supplementary material for: The predictive value of diaphragm ultrasound for weaning outcomes in critically ill children
Source: BMC Pulm Med. 2019 Dec 30;19:270. doi: 10.1186/s12890-019-1034-0 (PMC6937936; doi:10.1186/s12890-019-1034-0)
Supplement: Supplementary file 2 — Additional file 2: Table S1. linear correlation between DTF and PImax, DE [file 12890_2019_1034_MOESM2_ESM.docx]

**Supplemental Table 1. linear correlation between DTF and PImax, DE**

|  | DTF | |
| --- | --- | --- |
| Factors | *r* | *P* |
| DE | 0.380 | 0.006 |
| PImax | 0.410 | 0.003 |

**DTF** = diaphragmatic thickening fraction; **DE** = diaphragmatic excursion; **PImax** = maximum inspiratory pressure
